# Influence of physical training on erythrocyte concentrations of iron, phosphorus and magnesium

**DOI:** 10.1186/s12970-020-0339-y

**Published:** 2020-01-29

**Authors:** Marcos Maynar Mariño, Francisco Javier Grijota, Ignacio Bartolomé, Jesús Siquier-Coll, Victor Toro Román, Diego Muñoz

**Affiliations:** 1 Sport Sciences Faculty, University of Extremadura, Avenida de la Universidad s/n 10003, Cáceres, Spain; 2 Education Faculty, University of Extremadura, Avenida de la Universidad s/n 10003, Cáceres, Spain

**Keywords:** Iron, Magnesium, Phosphorous, Erythrocytes and training

## Abstract

**Background:**

The present study aimed to determine changes occurring in the erythrocyte concentrations of Iron (Fe), Magnesium (Mg) and Phosphorous (P) of subjects with different levels of physical training living in the same area of Extremadura (Spain).

**Methods:**

Thirty sedentary subjects (24.34 ± 3.02 years) without sports practice and a less active lifestyle, formed the control group (CG); 24 non-professional subjects (23.53 ± 1.85 years), who perform between 4 and 6 h/week of moderate sports practice without any performance objective and without following systematic training formed the group of subjects with a moderate level of training (MTG), and 22 professional cyclists (23.29 ± 2.73 years) at the beginning of their sports season, who performed more than 20 h/week of training, formed the high-level training group (HTG). Erythrocyte samples from all subjects were collected and frozen at − 80 °C until analysis. Erythrocyte analysis of Fe, Mg and P was performed by inductively coupled plasma mass spectrometry (ICP-MS). All results are expressed in μg/g Hb.

**Results:**

The results showed that there were statistically significant lower concentrations of erythrocyte Fe, Mg and P in MTG and HTG than CG. All parameters (Fe, Mg and P concentrations in erythrocytes) correlated inversely with physical training.

**Conclusions:**

Physical exercise produces a decrease in erythrocyte concentrations of Fe, Mg and P. This situation could cause alterations in the performance of athletes given the importance of these elements. For this reason, we recommend an erythrocyte control at the beginning, and during the training period, to avoid harmful deficits.

## Introduction

The concentration of mineral elements is usually under strict homeostatic control; however, physical activity alters this mechanism and brings changes in their serum levels [1–4]. Some studies report on the mineral concentrations in plasma, serum and urine. However, very few studies show the concentrations of the elements in the cellular compartment, and even less report on the influence of physical exercise on the cellular level of the mineral elements.

Fe is present mainly in the form of three proteins, hemoglobin in the red blood cells, myoglobin found in muscle cells and mitochondrial cytochromes [5, 6]. The deficiency of hemoglobin iron causes a decrease in oxygen transport to exercising muscles, thereby reducing physical work performance. Also, the deficit of non-heme Fe, which constitutes only around 1% of total body iron, may have detrimental effects for performance.

Often, these deficiencies have been observed by assessing indirect markers of iron concentration in the body such as the number of red blood cells, hemoglobin, hematocrit, ferritin and / or transferrin [7, 8]. However, we have not found studies showing these low intracellular concentrations of Fe.

Mg is the second most common intracellular cation, a mineral that acts in numerous metabolic processes related to physical activity [9, 10], and that also has a fundamental role as a cofactor in more than 300 enzymes involved in energy metabolism [10, 11]. Adequate body values of Mg are essential in physical activity, and a fall in body Mg can induce a drop in exercise performance, and, in the worst cases can lead to inflammatory responses, and an increase in oxidative stress [12]. So, adequate body Mg content can be critical for physical activity. Mg depletion can be caused by inadequate intake, excessive alcohol intake and increased sweating rates during exercise [13, 14]. It is usually evaluated by plasma and/or serum concentrations.

Phosphorus (P) is one of the most abundant mineral in the body, and plays an essential role in several aspects of cellular metabolism, including adenosine triphosphate (ATP) synthesis, which is the source of energy for many cellular reactions, and 2, 3-diphosphoglycerate concentration, which regulates the dissociation of oxygen from hemoglobin [15, 16]. Three major mechanisms are responsible for the maintenance of systemic phosphate homeostasis: intestinal uptake, retention or release from the bone, and renal reabsorption. Phosphorus also is an essential component of phospholipids in cell membranes. Changes in phosphorus content, concentration, or both, modulate the activity of some metabolic pathways [16, 17]. Like Mg, the concentrations of this mineral are measured in plasma or serum. Maynar-Mariño et al. [18] observed lower values of Mg and P in athletes than sedentary subjects, but it is unknown how these concentrations can affect intracellular values of these elements. Due to the deficiencies found in the extracellular compartment, it may be very important to observe the concentrations of these elements in erythrocytes, and the influence of physical activity on these values.

Therefore, it is essential to evaluate the influence of physical activity on the erythrocyte concentrations of several mineral elements that are involved in critical functions of the body during exercise. There are several studies about the acute effect of these minerals [19–21], but the effect of physical training on these elements in erythrocytes is still incomplete. What values of these mineral elements are found inside the cells of subjects who perform physical activity? To answer this question, the objective of this study was to evaluate the erythrocyte concentrations of Fe, Mg and P, in subjects who do not perform physical activity, in subjects who perform exercises of moderate intensity and in high-level training athletes.

## Materials and methods

### Participants

Thirty sedentary subjects, with an age of 24.34 ± 3.02 years, without sports practice and a less active lifestyle formed the control group (CG). Twenty-four non-professional subjects with an age of 23.53 ± 1.85 years, who perform between 4 and 6 h/week of moderate sports practice without any performance objective that imply an active lifestyle, without following any systematic training formed the group of subjects with a moderate degree of training (MTG). Twenty-two high-level athletes, professional cyclists at the beginning of their sports season, with an age of 23.29 ± 2.73, who performed more than 20 h/week of training, formed the high-level training group (HTG). On the basis of the total of hours/week of training, the subjects were classified into three categories: low (CG), moderate (MTG) and high (HTG).

Each participant had to satisfy the following criteria in order to be included in our study: to be male, non-smokers and not to have any health problems. The participants could not take any vitamins, minerals or other supplements during the study.

They were informed about the aim and procedures of the study, gave their informed consent and participated voluntarily. The University of Extremadura Ethics Committee approved the investigation according to the latest version of the Helsinki declaration for human research.

### Anthropometric measurements

The morphological characteristics of the participants were measured in the morning and always at the same time and in identical conditions. Body height was measured to the nearest 0.1 cm using a wall-mounted stadiometer (Seca 220. Hamburg. Germany). Body weight was measured to the nearest 0.01 kg using calibrated electronic digital scales (Seca 769. Hamburg. Germany) in nude, barefoot conditions. Body fat content was estimated from the sum of 6 skinfolds (∑6) (abdominal, suprailiac, tricipital and subscapularis, thigh and calf skinfolds). The skinfold thicknesses were measured with a Harpenden calliper (Holtain Skinfold Caliper. Crosswell, UK). All measurements were made by the same operator, skilled in kinanthropometric techniques, by the International Society for the Advancement of Kinanthropometry recommendations. All measurements were taken on the right side of the subject’s body. Heart rate and blood pressure were determined using an automatic sphygmomanometer (Omron HEM-780. Osaka. Japan) by a skilled technician, always after a five-minute rest period in the supine position.

### Nutritional evaluation

To guarantee they were following a similar diet, all participants completed a dietary questionnaire. The questionnaire consisted of a 3-day daily nutritional record, on two pre-assigned weekdays and one weekend day. On each day, participants individually indicated the type, frequency and quantity (in grams) of every food consumed, then the nutritional composition of their diets was evaluated using different food composition tables [22–24].

### Incremental test until exhaustion

An exercise test was used to evaluate the performance variables. The test consisted of a progressive load until exhaustion, on a cycle ergometer (Ergoline 900; Bitz, Germany) equipped with a gas analyzer (Metamax. Cortex Biophysik. Gmbh. Germany) and a Polar pulsometer (Polar. Norway).

Depending on the degree of training, two different protocols were used. The effort protocol used for the HTG consisted of 1 min entirely at rest, 15 min of warm-up, ending with 5 min at 100 watts; then starting at 150 watts and increasing the intensity by 25 watts every 3 min until reaching the maximum power they could maintain. In the case of MTG and CG, it consisted of 1 min entirely at rest, 15 min of warm-up ending with 5 min at 40 watts; then starting at 50 watts and increasing the intensity by 25 watts every 3 min until reaching the maximum power they could maintain. All tests were carried out under similar atmospheric conditions (21–24 °C and 45–55% relative humidity and atmospheric pressure between 700 and 715 mmHg).

The choice of these protocols was based on previous studies in which a slight increase in intensity was recommended for each step [25] and an adequate duration of the test (until exhaustion) to obtain VO_2_ max [26], as well as an adaptation based on the subject’s training level. Therefore, although starting with different loads, all the groups would face tests of similar duration and with the same increase in intensity [27]. The test was carried out on a cycle ergometer because of the greater accessibility for the collection of blood samples during the trial.

Training intensity and volume were reduced the two previous days applying a regenerative load to avoid fatigue in the test.

### Sample collection

#### Blood samples

After a fasting period of 8 h and before the test, 5 mL of venous blood was extracted from the antecubital vein of each participant using a plastic syringe fitted with a stainless-steel needle. Once extracted, the samples were collected into a metal-free polypropylene tube (previously washed with diluted nitric acid) with EDTA as anticoagulant. The blood samples were immediately centrifuged for 10 min at 3000 rpm. The plasma was separated, and the erythrocytes were washed with 0.9% sodium chloride (NaCl) three times. The erythrocytes were aliquoted into Eppendorf tubes (previously washed with diluted nitric acid) and conserved at − 80 °C until biochemical analysis.

##### Determination of hematocrit and hemoglobin

The hematocrits were obtained by centrifuging the whole blood into a glass capillary containing heparin in a Microcen microfuge (Alresa. Spain). Hemoglobin (Hb) was determined using a Hb analyzer (HemoCue. Sweden).

#### Erythrocytes elements determination

##### Sample preparation

The analysis was performed by inductively coupled plasma mass spectrometry (ICP-MS). To prepare the analysis, the decomposition of the organic matrix was achieved by heating it for 10 h at 90 °C after the addition of 0.8 mL HNO_3_ and 0.4 mL H_2_O_2_ to 2 mL of serum samples. The samples were then dried at 200 °C on a hot plate. Sample reconstitution was carried out by adding 0.5 mL of nitric acid, 10 μL of Indium (In) (10 mg/L) as an internal standard, and ultrapure water to complete 10 mL.

##### Standard and reference material preparation

Reagent blanks, element standards and certified reference material (Seronorm, lot 0511545, Sero AS Billingstand, Norway) were prepared identically and used for accuracy testing. Before the analysis, the commercial control materials were diluted according to the manufacturer’s recommendation.

##### Sample analysis

Digested solutions were assayed by an ICP-MS Nexion model 300D (PerkinElmer, Inc., Shelton, CT, USA) equipped with a triple quadrupole mass detector and a reaction cell/collision device that allows operation in three modes: without reaction gas (STD); by kinetic energy discrimination (KED) with helium as the collision gas; and in reaction mode (DRC) with ammonia as the reaction gas. Both collision and reaction gases such as plasmatic argon had a purity of 99.999% and were supplied by Praxair (Madrid, Spain). Two mass flow controllers regulated gas flows. The frequency of the generator was free-swinging and worked at 40 Mhz. Three replicates were analyzed per sample. The sample quantifications were performed with indium (In) as an internal standard. The values of the standard materials of each element (10 μg/L) used for quality controls were in agreement with intro and inter-assay variation coefficients of less than 5%.

### Statistical evaluations

Statistical analyses were carried out with the SPSS 20.0 for Windows. The results are expressed as *x* ± *s*, where *x* is the mean value and *s* the standard deviation.

The Dixon Q test was used to identify outliers. These values were analyzed to evaluate if their magnitude warranted their elimination from the analyses. Afterward, an exploration of the different variables was carried out to determine normality, using the Shapiro-Wilks test, recommended for samples of less than 30 individuals. Subsequently, a comparison of the behavior of the variables among the three groups was made, using an ANOVA test, and applying a Bonferroni test later on if there was significance.

A Pearson correlation study was carried out to ascertain if there was a relationship between erythrocyte changes in the concentrations of the elements and physical training. A significant difference was considered when *p* < 0.05.

## Results

Table 1, shows the anthropometric data of CG, MTG and HTG. As can be observed, the significantly decreased total weight and body fat percentage in MTG and HTG, indicate the adaptive consequences of training.

**Table 1:** Characteristics of the three groups in the study

	CG (*n* = 30)	MTG (*n* = 24)	HTG (*n* = 22)
Height (m)	1.75 ± 0.18	1.77 ± 0.05	1.73 ± 0.07
Weight (kg)	75.34 ± 12.03	75.12 ± 8.16	65.61 ± 5.22^†*^
Σ6 skinfolds (mm)	110.61 ± 34.29	87.78 ± 30.14	56.32 ± 16.65^†*^
VO_2Máx_ (mL·min^− 1^·kg^− 1^)	38.90 ± 3.73	46.77 ± 6.72^++^	61.23 ± 3.00^††**^
VE_max_ (L/min)	89.33 ± 12.31	121.11 ± 21.40^++^	153.17 ± 15.84^††**^
HR_basal_ (beats·min^− 1^)	72.58 ± 7.38	54.90 ± 10.85^++^	62.85 ± 7.57^††**^
HRmax (beats·min^− 1^)	184.32 ± 12.71	194.96 ± 8.3^+^	196.71 ± 6.21^†^

Anova and Bonferroni tests

† Differences between the HTG and CG (†*p* < 0.05; ††*p* < 0.01)

+ Differences between the MTG and CG (+*p* < 0.05; ++*p* < 0.01)

* Differences between the HTG and MTG (*p < 0.05; **p < 0.01)

The results of some ergoespirometric parameters are also shown. A significant increase in both training groups, can also be observed as would be expected. The data correspond to high endurance intensity training athletes and subjects with a medium and normal condition. Maximal VO_2_ and VE were significantly higher in the two training groups than controls. Maximal HR was lower in the control group than the training groups, and basal HR was lower in the training groups than the controls.

Table 2, presents the daily intake of Fe, Mg and P. The results are presented in mg/d. No differences among groups were found.

**Table 2:** Daily intake of Fe, Mg and P in CG and sportsmen classified by the level of training

	CG (*n* = 30)	MTG (*n* = 24)	HTG (*n* = 22)
Fe (mg/d)	15.91 ± 4.62	16.04 ± 4.22	16.78 ± 5.34
Mg (mg/d)	323.89 ± 78.34	331.24 ± 77.64	337.54 ± 98.92
P (mg/d)	764.22 ± 110.60	758.32 ± 123.67	743.33 ± 156.8

Table 3 shows the results for hemoglobin and hematocrit. Both parameters were similar in the three groups.

**Table 3:** Hemoglobin and hematocrit values in CG and sportsmen classified by the level of training

	CG (*n* = 30)	MTG (*n* = 24)	HTG (*n* = 22)
Hemoglobin (g/dl)	14.97 ± 1.01	15.46 ± 1.45	15.14 ± 0.84
Hematocrit (%)	41.31 ± 1.18	42.89 ± 1.12	43.32 ± 1.17

Anova and Bonferroni tests

Table 4 presents the erythrocyte concentrations of Fe, Mg and P. The results are presented in μg/gHb, given that the major protein in the erythrocyte is hemoglobin and thus the results obtained in all cases are more solid.

**Table 4:** Concentrations of Fe, Mg and P in CG and sportsmen classified by the level of training

	CG (*n* = 30)	MTG (*n* = 24)	HTG (*n* = 22)
Fe (μg/gHb)	907.78 ± 152.00	659.50 ± 211.66^+++^	523.96 ± 79.28†††*
Mg (μg/gHb)	38.69 ± 6.88	27.35 ± 9.25^+++^	23.52 ± 4.09†††
P (μg/gHb)	546.18 ± 94.50	436.12 ± 121.78^+++^	405.20 ± 44.87†††

Anova and Bonferroni tests

† Differences between the HTG and CG (†*p* < 0.05; ††*p* < 0.01; †††*p* < 0.001)

+ Differences between the MTG and CG (+*p* < 0.05; ++*p* < 0.01; +++*p* < 0.001)

* Differences between the MTG and HTG (*p < 0.05; **p < 0.01; ***p < 0.001)

Fe, Mg and P concentrations were lower in MTG and HTG (*p* < 0.001) than CG. GMT presented a higher concentration of Fe than HTG (*p* < 0.05).

Table 5, showed the correlations between the three elements and training. Results are expressed with a correlation coefficient (**r**) and with significance level (**p**). We found that the erythrocyte concentrations of Fe, Mg and P showed a higher (*p* = 0.000) correlation with training.

**Table 5:** Correlations among the 76 subjects, represented by the r; statistical significance, Fe, Mg and P and the level of training

Element	Training status
Fe	−0.744; *p* = 0.000
Mg	−0.695; *p* = 0.000
P	−0.568; *p* = 0.000

## Discussion

As previously mentioned, possible deficiencies in Fe, Mg and P are usually evaluated in plasma or serum, but not in the intracellular compartment. Due to the importance of these elements for cell functions, it is necessary to know what occurs in the intracellular compartment when a deficiency is observed in the extracellular one (a balance between both compartments is required to maintain proper cell function). If a difference were observed, the cell functions could be affected.

In the present study, we evaluated the concentrations of Fe, Mg and P in erythrocytes of different subject groups (CG, MTG, HTG) with the aim of reflecting the cell concentrations. The analysis of elements in erythrocytes has many advantages. Whole blood (and consequently, red blood cells) are readily available; the lifetime of erythrocytes, which is 120 days, can give us retrospective information about their deposits. Also, the concentration in erythrocytes is not subject to transient variations such as those found in plasma or serum.

We used three well-differentiated groups for this study, as is reflected in Table 1. The effect of the regular practice of physical exercise can be observed in the MTG and HTG groups and the cardiorespiratory and anthropometric adaptations.

No significant changes were found when evaluating the results of the daily intake of the elements.

When evaluating the results of this study, we found that the subjects who exercised regularly presented significantly lower erythrocyte concentrations of Fe, Mg and P, than those subjects who did not exercise regularly and that these concentrations were correlated with the training.

Fe deficiency is the most common nutritional deficiency in the world, even in the wealthiest countries [28]. Athletes, particularly women and adolescents, are at an increased risk of depleting their Fe deposits to a state of functional or absolute deficiency that, if not recognized or treated, can develop into sideropenic anemia [29]. When Fe deposits are inadequate, physical performance may decrease, presenting fatigue, intolerance to exercise and various cognitive impairments [30–32].

Drops in Fe can result from some clinical and pathological conditions, hemorrhages, peptic ulcer, stomach cancer and ulcerative colitis. In general, apart from the Fe losses due to sweating, which can be considerable [33], there must be other Fe requirements that are associated with changes in blood levels. For example, athletes require high intakes of Fe because of their larger volume of blood, gastrointestinal bleeding and hemolysis that occur due to stress and repeated damage [34, 35]. Furthermore, during the performance of physical exercise, an alarming increase in the expression of hepcidin has been seen as a result of a negative balance of Fe in the athletes [36].

Our study showed significantly lower Fe erythrocyte concentrations in the two groups of athletes (MTG and HTG) than CG, although there were no significant differences in the Hb of the different groups. Moreover, the lowest Fe concentrations were found in the HTG.

The concentrations of Fe in CG were similar to those recently presented by Lu et al. [37] with the same technique as in our study. However, MTG and HTG presented lower values than CG in this parameter. These results could indicate a Fe deficiency in high-level athletes’ erythrocytes, which could have a negative consequence in relation to oxygen transport and performance.

Fe had a very significant inverse correlation (*r* = − 0.744, *p* < 0.001) with training degree, with lower values the more trained the subjects, which would indicate that this deficit could be due to the changes produced by intense training. These low values would probably be related to deficiencies in iron, as some studies have indicated [36, 38, 39], produced by the same mechanisms as previously mentioned.

The diagnosis of Mg deficiencies is problematic because low plasma Mg concentrations may occur in patients with normal intracellular concentrations and pronounced intracellular deficiencies can occur with normal plasma values [40–42].

Maynar et al. [43] reported that physical exercise can influence the serum concentration of magnesium in sports people. One of the most common findings, in some investigations, is a decrease in plasma magnesium levels after physical exercise [44]. Also, a plasma and serum decrease in Mg levels has been observed when studying the effects of the practice of long-term endurance exercise (marathon or cross-country skiing) [45, 46]. Several studies have indicated that athletes are deficient in Mg [13, 47]. Maintaining adequate concentrations of magnesium is necessary for athletes to sustain an appropriate level of athletic performance given the importance of this element in the use of high energy molecules, in muscle contraction and in maintaining the properties of cell membranes [48]. Thereby, an alternative method for estimating the Mg store includes direct measurements of intracellular Mg using skeletal muscle [49], erythrocyte [50, 51], or lymphocytes [52]. The erythrocyte concentration of Mg has become popular in the evaluation of body status of Mg [41, 53–55].

In the current study, the erythrocyte concentration of Mg was significantly lower in HTG and MTG than CG, which would be related to the lower serum concentrations found by Maynar-Mariño et al. (2015) in athletes. Recent research obtained similar results during a cycling race [44]. There is also a very high correlation with the degree of training as shown in Table 5. Given the importance of the intraerythrocytic concentration of Mg in body levels, the results indicate that our athletes had a deficit in Mg, as Maynar-Mariño et al. [18] reported in high level athletes, using the same technique in serum. This could reduce their performance, given the importance of this element as discussed above. In the same way as Fe, there was a very high correlation of Mg with training level. Therefore, physical training would be an important factor involved in the erythrocyte values of Mg. On the other hand, there is greater sweating in athletes, which could lead to this situation since this element is eliminated in this way. Also, another possible loss would be because of a redistribution of Mg during exercise to tissues and cells, because different studies confirm that a magnesium flow occurs during and after aerobic physical exercise [13, 56].

P is necessary for a multitude of reactions in which energy is required, being basic in the production of energy molecules such as adenosine triphosphate (ATP), creatine phosphate and phosphoenolpyruvic acid. It also contributes to the control of the acid-base balance in the blood.

In our study, we found, as in the case of Fe and Mg, that erythrocyte concentrations were significantly lower in subjects who practiced physical activity than in the CG and are inversely correlated with the athletes’ degree of training (*p* < 0.001; *r* = − 0.568), as was the case with Mg. So, the subjects with a higher level of training present lower concentrations of P. Maynar-Mariño et al. (2015) observed significantly lower serum concentrations in athletes of high regional level compared to CG [43]. Therefore, our study suggests decreased concentrations in the intracellular compartment in trained subjects too, which could reflect alterations in cell functions, including myopathy, ultrastructural changes and skeletal muscle injuries [57, 58].

The causes of this decline in the elements studied in athletes could be: a deficient intake of these in the diet of the athlete or overhydration in the subjects who perform training as a known mechanism to this effect occurs in the initial phases of physical training in aerobic athletes. However, the lower levels of these elements in athletes could not be due to a deficit in the diet since there were no differences between groups in the intake of these metals. Nonetheless, hyperhydration in the cellular compartment would lead to a higher dilution of the elements contained in the erythrocytes and a lower concentration. Previous research shows intracellular deficits of Fe, Mg and P, related to those found in serum by Maynar-Mariño et al. [18]. Additionally, recent research observed a drop in baseline erythrocyte concentration of Mg in two groups (with and without supplementation of Mg) during a professional cyclist race [44]. Besides, the mentioned paper reported that there is a greater release of erythrocyte Mg in order to alleviate the oxidative stress caused by exercise. Elsewhere, the redistribution of blood during exercise could decrease the blood flow to the intestine and impair the absorption of these elements [59, 60].

In relation to Fe, many deficits are known, evaluated with related parameters, like hematocrit or hemoglobin or ferritin. We only determined the hematocrit and hemoglobin concentration, with similar results in both groups. However, we cannot affirm that an extracellular deficiency was present, because we did not obtain the concentration of plasmatic ferritin. But a low concentration of Fe was obtained in the erythrocytes because of the physical training,

For this reason, we believe that it is necessary to carry out studies in which this phenomenon can be taken into account [61].

## Conclusions

In conclusion, our study reveals an erythrocyte deficiency in Fe, Mg and P in subjects who perform physical training, which does not exist in subjects who do not practice regular exercise, and these deficiencies are correlated with sports training. Therefore, we believe that the cell evaluation of Fe, Mg and P should be performed in athletes who perform systematic training before and during their training phase to detect early any deficiency of these elements that could lead the athlete to a decrease in performance.

## Data Availability

All data generated or analyzed during this study are included in this published article.

## Abbreviations

2,3 DFG: 2,3-diphosphoglycerate
ATP: Adenosine triphosphate
CG: Control group
Fe: Iron
H_2_O_2_: Hydrogen peroxide
Hb: Hemoglobin
HR: Heart rate
HTG: High training group
ICP-MS: Inductively coupled plasma mass spectrometry
IPAQ: International Physical Activity Questionnaire
MET: Metabolic equivalent task
Mg: Magnesium
MTG: Moderate training group
P: Phosphorus
VE: Expiratory Volume
VO_2_: Oxygen Consumption
Zn: Zinc
Σ6: Sum of 6 skinfolds
