# Correction to: Influence of physical training on erythrocyte concentrations of iron, phosphorus and magnesium

**DOI:** 10.1186/s12970-020-00363-8

**Published:** 2020-06-30

**Authors:** Marcos Maynar Mariño, Francisco Javier Grijota, Ignacio Bartolomé, Jesús Siquier-Coll, Victor Toro Román, Diego Muñoz

**Affiliations:** 1 Sport Sciences Faculty, University of Extremadura, Avenida de la Universidad s/n, 10003 Cáceres, Spain; 2 Education Faculty, University of Extremadura, Avenida de la Universidad s/n, 10003 Cáceres, Spain

**Correction to: J Int Soc Sports Nutr 17, 8 (2020)**

**https://doi.org/10.1186/s12970-020-0339-y**

The original article [1] contains an error in Table 4 whereby values are incorrectly displayed due to a misplaced separator. The correct version of Table 4 can be viewed ahead in this Correction article.

**Table 4:** Concentrations of Fe, Mg and P in CG and sportsmen classified by the level of training

	CG (*n* = 30)	MTG (*n* = 24)	HTG (*n* = 22)
**Fe (**mg/gHb**)**	9.07 ± 1.52	6.59 ± 2.11^+++^	5.23 ± 0.79†††*
**Mg (**μg/gHb**)**	386.9 ± 68.8	273.5 ± 92.5^+++^	235.2 ± 40.9†††
**P (**mg/gHb**)**	5.46 ± 0.94	4.36 ± 1.21^+++^	4.05 ± 0.44†††

Anova and Bonferroni tests

† Differences between the HTG and CG (†*p* < 0.05; ††*p* < 0.01; †††*p* < 0.001)

+ Differences between the MTG and CG (+*p* < 0.05; ++*p* < 0.01; **+++***p* < 0.001)

* Differences between the MTG and HTG (*p < 0.05; **p < 0.01; ***p < 0.001)
